# Kaempferol Alleviates Aflatoxin B1-Induced Liver Injury by Mitigating Oxidative Stress

**DOI:** 10.3390/nu18162633

**Published:** 2026-08-12

**Authors:** Zongmin Shu, Qingyi Zhou, Mao Zhu, Lan Yang, Yujie Chen, Yongyun Zhang, Junlong Bi, Weizhen Li, Ming Li

**Affiliations:** 1 College of Veterinary Medicine, Yunnan Agricultural University, Kunming 650201, China; 18387175872@163.com (Z.S.); z3307329523@163.com (Q.Z.); zhumao25@mails.ucas.ac.cn (M.Z.); 19187179246@163.com (L.Y.); cyj619841703@126.com (Y.C.); junlongbi@foxmail.com (J.B.); 2 Teaching Demonstration Center of the Basic Experiments of Agricultural Majors, Yunnan Agricultural University, Kunming 650201, China; zyypang@126.com; 3 Kunming Institute of Zoology, Chinese Academy of Sciences, Kunming 650201, China

**Keywords:** AFB1, kaempferol, Keap1/Nrf2, oxidative stress, network pharmacology

## Abstract

**Background** Aflatoxin B1 (AFB1) is a potent hepatotoxic mycotoxin that induces severe oxidative liver damage. Kaempferol (Kae), a natural flavonoid with known antioxidant properties, has unclear protective effects against AFB1-induced hepatotoxicity. This study aimed to evaluate the hepatoprotective role of Kae and elucidate its underlying mechanism using integrated in vivo, in silico, and in vitro approaches. **Methods**: In vivo (AFB1-challenged mice) and in vitro (hepatocyte) models were employed, combined with network pharmacology, molecular docking, and molecular dynamics simulations. Liver injury indices, oxidative stress markers, antioxidant enzyme activities, and Keap1/Nrf2 pathway expression were assessed. **Results**: Kae co-treatment reversed AFB1-induced increases in liver index, serum ALT/AST, histological lesions, and reduced antioxidant capacity in mice. Network pharmacology revealed 59 common targets, with NFE2L2 (Nrf2) as a key node. In vitro, Kae pretreatment significantly lowered AFB1-elevated ROS, MDA, ALT, and AST, while restoring GSH and total antioxidant capacity. Kae reversed AFB1-induced Keap1 upregulation and Nrf2 downregulation, and increased mRNA levels of *HO-1*, *NQO1*, *SOD*, *GPX1*, and *CAT*. Molecular docking and simulation showed stable Kae–Keap1 binding (−9.6 kcal/mol) with critical hydrogen bonds (VAL-606) and van der Waals contacts. **Conclusions**: Kae directly binds Keap1, activates Nrf2 signaling, upregulates antioxidant gene expression, and mitigates AFB1-induced oxidative liver injury. These findings support Kae as a promising candidate for preventing AFB1 hepatotoxicity.

## 1. Introduction

AFB1, the most potent member of the aflatoxin family (B1, B2, G1, G2, M1, M2), is a difuranocoumarin mycotoxin biosynthesized predominantly by A. flavus and A. parasiticus under warm, humid conditions [1]. Its extreme hepatotoxicity and potent carcinogenicity rank it among the most hazardous contaminants in the global food chain [1,2]. AFB1 frequently contaminates moldy feed and food, causing toxic effects in mammals, birds, fish, and other animals. It poses serious risks to global health and leads to considerable economic losses [3]. Chronic AFB1 intake reduces growth performance in poultry, evidenced by lower daily weight gain, decreased feed intake, and poorer feed conversion. It also compromises immune function and antioxidant defenses, resulting in immune organ damage, lower antibody titers, and vaccine failure. Moreover, pathological changes occur in the liver, intestines, and kidneys [4]. Serving as the principal hub for xenobiotic biotransformation, the liver is also the primary target of AFB1-induced cytotoxicity. Exposure to this potent mycotoxin initiates a continuum of hepatocellular damage, ranging from reversible degeneration and necrotic cell death to apoptosis. When injury persists whether from chronic dietary exposure or acute high-concentration intoxication maladaptive repair processes promote cirrhosis and ultimately drive hepatocarcinogenesis [5]. In hepatocytes, AFB1 is metabolized by cytochrome P450 enzymes to the reactive intermediate aflatoxin B1-8,9-epoxide (AFBO). This epoxide binds to lysine ε-amino groups, forming adducts that are unstable and hydrolyze to AFB1 dihydrodiol. The dihydrodiol then reacts with various proteins, inducing tissue damage, inflammation, and abnormal cell proliferation, which can promote cancer [6]. The flavonoid hesperidin has been shown to protect African catfish against AFB1-induced oxidative stress, hepatic and renal dysfunction, neurotransmitter disturbances, and histological alterations, thereby contributing to more sustainable aquaculture practices [7]. Overall, AFB1 exerts multi-organ toxicity-particularly in the liver-through its metabolic activation.

Redox homeostasis collapse driven by AFB1 unleashes uncontrolled accumulation of reactive intermediates, initiating lipid peroxidation cascades that culminate in hepatocellular injury [8]. Since oxidative stress is a central mechanism of AFB1 hepatotoxicity [9], bolstering endogenous antioxidant defenses represents a rational interventional strategy. This rationale has spurred intensive interest in phytochemically derived bioactives, which offer multi-target modulatory advantages over synthetic antioxidants. Preclinical evidence confirms that astaxanthin concurrently engages the Nrf2/Keap1 cytoprotective axis and suppresses NF-κB-mediated inflammation, synergistically restoring SOD and GSH pools while attenuating ROS and MDA accumulation to reverse histopathological lesions [10]. Phlorotannins from brown algae mitigate AFB1-evoked oxidative stress and mitochondrial dysfunction in broilers via sustained Nrf2 activation [11]. Dandelion sterols relieve AFB1-induced oxidative stress and hepatocyte apoptosis by upregulating antioxidant enzyme expression and inhibiting CYP1A1 activity [12]. Therefore, developing natural compounds capable of alleviating oxidative stress is crucial for mitigating AFB1-induced liver injury.

Kaempferol (Kae, 3,5,7-trihydroxy-2-(4-hydroxyphenyl)-4H-chromen-4-one) is a natural flavonoid abundant in tea, broccoli, and citrus fruits [13]. Kae exhibits a broad spectrum of pharmacological activities, including anticancer, antioxidant, antiviral, anti-inflammatory, antibacterial, and immunomodulatory effects, as reported in previous studies [14]. These properties confer considerable antioxidant activity to Kae, suggesting its potential as a candidate for drug development. Kae exerts distinct therapeutic functions against liver diseases induced by different etiological factors. In a mouse study on alcoholic liver injury, Kae reduced CYP2E1 activity, which led to less ROS overproduction during ethanol breakdown and also boosted the body’s own antioxidant defenses [15]. It has been found that in an APAP-induced liver injury model, Kae significantly boosts hepatic GSH and SOD activities, curbs MDA and ROS accumulation, and offers hepatoprotection through the reduction in oxidative stress and improvement of mitochondrial dysfunction [16]. With respect to anti-inflammatory actions, Kae inhibits osteoclastogenesis by activating the Nrf2/HO-1 axis, thereby offering therapeutic benefits in inflammatory osteolytic diseases [17]. Moreover, Kae attenuates T-cell immunosenescence and inflammatory responses via activation of the SIRT3-LKB1-AMPK-mitophagy axis [18]. The ability of Kae to prevent abdominal aortic aneurysm is mediated through the modulation of macrophage polarization and STAT/TNF signaling [19]. It has been demonstrated that MOLE alleviates AFB1 hepatotoxicity by activating the PPARγ/Nrf2 pathway, with Kae identified as a primary active ingredient [20]. Although Kae serves as one of the key bioactive constituents of MOLE, how it mitigates AFB1-induced liver injury has yet to be elucidated.

While previous studies have reported kaempferol’s capacity to alleviate oxidative liver damage, the molecular details underlying its protection against AFB1 toxicity remain incompletely understood. Therefore, this study aimed to investigate the ameliorative potential of Kae against AFB1 hepatotoxicity using in vivo experiments, network pharmacology, in vitro validation, and molecular docking/simulations. Our results complement existing phenotypic evidence by clarifying the potential structural basis of kaempferol-mediated Keap1/Nrf2 pathway activation, offering a more complete framework for understanding its protective mechanisms.

## 2. Materials and Methods

All animal experiments and cell-based procedures were conducted in accordance with relevant guidelines and regulations. A detailed study protocol, including the research question, key design features, and statistical analysis plan, was prepared prior to commencing the experiments. Detailed protocols supporting the findings of this study are described below. All relevant materials, raw data, and protocols are available from the corresponding author upon reasonable request. No generative artificial intelligence was employed in the design, execution, analysis, or interpretation of this study; only automated tools for superficial language editing (i.e., grammar and spelling checks) were utilized.

### 2.1. Animal Experiment Design

Given that hepatic AFB1 metabolism and Keap1/Nrf2-mediated antioxidant responses are well-established and conserved across mammals including humans, C57BL/6 mice were used to evaluate kaempferol’s protection against AFB1 hepatotoxicity. SPF-grade male C57BL/6 mice (18–22 g body weight) were procured from the Laboratory Animal Center of Yunnan University (Kunming, China, Production License No. SCXK-K2022-0002). The experimental unit for all in vivo analyses was an individual mouse. Mice were maintained in a barrier unit under a 12 h light/dark cycle, with environmental enrichment provided, and ad libitum access to feed and water. Group sizes (*n* = 6 per cohort) were predefined via a priori power analysis (α = 0.05, statistical power = 0.8) implemented in G*Power 3.1, cross-validated against pilot experimental data to guarantee adequate statistical reliability.

After a 7-day acclimation period, study mice were randomized into three cohorts (*n* = 6 per cohort) via a computer-generated pseudorandom allocation schedule. The experimental groups were: (1) Control group: received 0.5% carboxymethylcellulose (CMC) via oral gavage; (2) AFB1 group: received 0.75 mg/kg AFB1 dissolved in 0.5% CMC daily; (3) AFB1 + Kae group: received 100 mg/kg Kae plus 0.75 mg/kg AFB1 daily for 30 days. The AFB1-induced hepatotoxicity model was established as described previously [21]. The human equivalent dose of 100 mg/kg Kae was calculated as approximately 8.1 mg/kg based on the body surface area normalization method. All experimental procedures were conducted by investigators who were blinded to the group allocations.

During the study, healthy mice with stable post-acclimation weight (no exclusions) were monitored daily for clinical signs. Humane endpoints (>20% weight loss or severe lethargy) triggered immediate euthanasia via intraperitoneal sodium pentobarbital injection per AVMA Guidelines. Procedures were performed under isoflurane anesthesia to minimize distress, and no unexpected adverse events occurred. Upon reaching the prespecified terminal endpoint of the experimental period, all enrolled mice were subjected to inhalational anesthesia using isoflurane vapor, followed by terminal whole-blood collection and hepatic tissue harvest for downstream biochemical and molecular analyses. Prior ethical clearance for all study procedures was granted by the Animal Ethics Committee of Yunnan Agricultural University (Protocol Approval No. 202303008; issued 8 March 2023). 

### 2.2. Liver Index, Histology, and Biochemical Assays

The hepatosomatic index was calculated as (liver weight/body weight) × 100%. For histological assessment, liver sections were prepared via standard H&E staining protocols and examined under a light microscope. Serum alanine aminotransferase (ALT) and aspartate aminotransferase (AST) were quantified using a fully automated biochemistry analyzer. Hepatic oxidative stress parameters, including malondialdehyde (MDA), reduced glutathione (GSH), and total antioxidant capacity (T-AOC), were measured in 5% tissue homogenates using commercial kits (Solarbio, Beijing, China; Cat. Nos. BC6415, BC1170, BC1315) according to the manufacturer’s instructions.

### 2.3. RT-qPCR

Total RNA was isolated from liver tissues or cultured cells via TRIzol™ reagent (Invitrogen, Carlsbad, CA, USA), with nucleic acid purity confirmed via A260/A280 absorbance ratios. First-strand complementary DNA (cDNA) was generated using a commercial reverse transcription kit, followed by quantitative real-time PCR (qPCR) amplification on a Bio-Rad MyiQ™ (Bio-Rad Laboratories, Hercules, CA, USA) thermal cycler with SYBR™ Green qPCR Master Mix. Amplification specificity for all target genes was validated via post-run melting curve profiling. Relative transcript abundances were normalized to the endogenous reference gene β-actin and quantified using the 2^−ΔΔCt algorithmic framework. All oligonucleotide primer sequences employed in this study are compiled in Table S1.

### 2.4. Network Pharmacology

Potential targets of kaempferol were predicted using Swiss Target Prediction (http://www.swisstargetprediction.ch/ (accessed on 15 November 2023)), PharmMapper (http://lilab-ecust.cn/pharmmapper/ (accessed on 15 November 2023)), and SEA (https://sea.bkslab.org (accessed on 15 November 2023)). AFB1 hepatotoxicity-related targets were retrieved from GeneCards (https://www.genecards.org (accessed on 20 November 2023)), OMIM (https://omim.org (accessed on 20 November 2023)), and MalaCards (https://www.malacards.org (accessed on 20 November 2023)). Overlapping targets between the two sets were identified using Venny and uploaded to STRING (https://string-db.org (accessed on 25 November 2023)) with a minimum confidence score of 0.4 to construct a protein–protein interaction (PPI) network. The network was visualized and analyzed in Cytoscape (v3.9.1); core targets were selected based on degree, betweenness, and closeness centrality calculated with the CentiScaPe 2.2 plugin. Gene Ontology (GO) and Kyoto Encyclopedia of Genes and Genomes (KEGG) enrichment analyses were performed using DAVID (https://david.ncifcrf.gov (accessed on 28 November 2023)), and results were plotted using the Weishengxin platform (https://www.bioinformatics.com.cn (accessed on 28 November 2023)).

### 2.5. Cell Culture and Treatments

Murine AML12 hepatocytes were propagated in RPMI 1640 medium supplemented with 10% (*v*/*v*) fetal bovine serum (FBS) plus 1% (*v*/*v*) penicillin-streptomycin, in a humidified incubator at 37 °C with 5% CO_2_. For experimental treatments, cells were administered a 1 h preconditioning dose of 10 µM kaempferol prior to challenge with 20 µM AFB1 for predefined experimental durations.

### 2.6. Cell-Based Assays

AML12 cells were maintained in 96-well formats for 24 h, by which point cellular confluence had reached 70–80% prior to viability assessment via the CCK-8 assay. Subsequent treatments with kaempferol (Kae; 0–200 μM) were then administered for 12 h ahead of AFB1 exposure Concentrations up to 100 µM and 200 µM significantly reduced cell viability (*p* < 0.05). Therefore, 10 μM Kae was selected for subsequent mechanistic assays as it provided optimal protection against AFB1-induced cytotoxicity within the non-toxic range. The levels of ALT and AST in cell culture supernatants were measured using an automatic biochemical analyzer. For intracellular ROS quantification, cultured AML12 hepatocytes were incubated with the cell-permeable fluorogenic 10 µM DCFH-DA probe for 30 min under dark, humidified conditions at 37 °C, with resultant fluorescence signals captured via an inverted epifluorescence microscope (Olympus, CKX53, Olympus Corporation, Tokyo, Japan). Levels of MDA, GSH, and T-AOC were quantified in sonicated whole-cell lysates using the identical validated commercial assay kits employed for the in vivo hepatic analyses. For Western blotting, cells were lysed in RIPA buffer containing protease and phosphatase inhibitors. Proteins were separated by SDS-PAGE, transferred to nitrocellulose membranes, blocked with protein-free blocking buffer, and incubated overnight with primary antibodies against Keap1 (cat. no. R26935, ZenBio Co., Ltd., Chengdu, China), Nrf2 (cat. no. BS1258, Bioworld Technology Inc., Bloomington, IN, USA), and β-actin (cat. no. AC026, ABclonal Biotechnology Co., Ltd., Wuhan, China). After incubation with horseradish peroxidase-conjugated secondary antibodies, signals were visualized with enhanced chemiluminescence (ECL) (Biosharp, BL520B, Hefei, China) and captured by a chemiluminescence imaging system (Servicebio, Wuhan, China). Protein loading was normalized using β-actin as an internal reference, and band intensities were quantified using ImageJ 1.54f software (U.S. National Institutes of Health, Bethesda, MD, USA).

### 2.7. Molecular Docking

The three-dimensional structure of kaempferol was obtained from PubChem (https://pubchem.ncbi.nlm.nih.gov (accessed on 5 December 2023)), and the crystal structure of Keap1 (PDB ID: 1ZGK) was downloaded from the Protein Data Bank (https://www.rcsb.org (accessed on 5 December 2023)). Molecular docking was performed using the CB-Dock2 web server (https://cadd.labshare.cn/cb-dock2/php/index.php (accessed on 5 December 2023)) with default parameters [22]. The best docking pose was selected based on binding energy and visualized using PyMOL 2.0 and Discovery Studio 2023.

### 2.8. Immunofluorescence Staining

Immunofluorescence staining was conducted on AML12 hepatocytes cultured on coverslips. Following fixation with 4% paraformaldehyde for 15 min and permeabilization with 0.2% Triton X-100, samples were incubated with 5% normal donkey serum for 30 min to block non-specific binding. Primary anti-Nrf2 antibody was applied overnight at 4 °C, followed by a 1 h incubation with Alexa Fluor-conjugated secondary antibody at room temperature. Nuclei were counterstained with DAPI, and cells were washed three times with PBS prior to mounting with antifade medium and visualization under an inverted fluorescence microscope.

### 2.9. Molecular Dynamics Simulation

Simulations were executed using GROMACS 2022.2, employing the Amber14SB force field and the TIP3P explicit water model for a total duration of 100 ns. To ensure electroneutrality, the system was neutralized with 0.15 M NaCl. Subsequently, the complex underwent steepest-descent energy minimization followed by a two-phase equilibration process—first under the canonical (NVT) ensemble and subsequently under the isothermal-isobaric (NPT) conditions at 298 K and 1 bar. Throughout the production run, long-range electrostatic interactions were computed using the particle mesh Ewald (PME) method, while short-range nonbonded forces were truncated at 1.2 nm via the Verlet cutoff scheme. Covalent bonds involving hydrogen were constrained using the LINCS algorithm. Finally, the gmx_MMPBSA toolkit was utilized to estimate the binding free energy.

### 2.10. Statistical Analysis

Data from in vitro experiments are presented as mean ± standard deviation (SD) from at least three independent experiments. For in vivo studies, data are presented as mean ± SD from six biological replicates (*n* = 6 mice per group). Prior to parametric testing, normality of data distribution was assessed using the Shapiro–Wilk test, and homogeneity of variance was evaluated using Levene’s test; Welch’s correction was applied for comparisons with unequal variances. Statistical comparisons among groups were performed using one-way analysis of variance (ANOVA) followed by appropriate post hoc tests, with GraphPad Prism10.1 (GraphPad Software, Boston, MA, USA) A *p* < 0.05 was considered statistically significant.

### 2.11. Data Availability Statement

All raw data generated during this study are available from the corresponding author upon reasonable request. No publicly archived datasets were used in this work.

### 2.12. Generative AI Statement

No generative AI or AI-assisted tools were used in the design, execution, data analysis, or interpretation of this study. The manuscript was subjected to superficial language editing (grammar and spelling) using Microsoft Word 2024 (Microsoft Corporation, Redmond, WA, USA), which does not require disclosure per the journal’s policy.

## 3. Results

### 3.1. Kae Alleviates AFB1-Induced Body Weight Loss and Liver Injury in Mice

**Figure 1:**
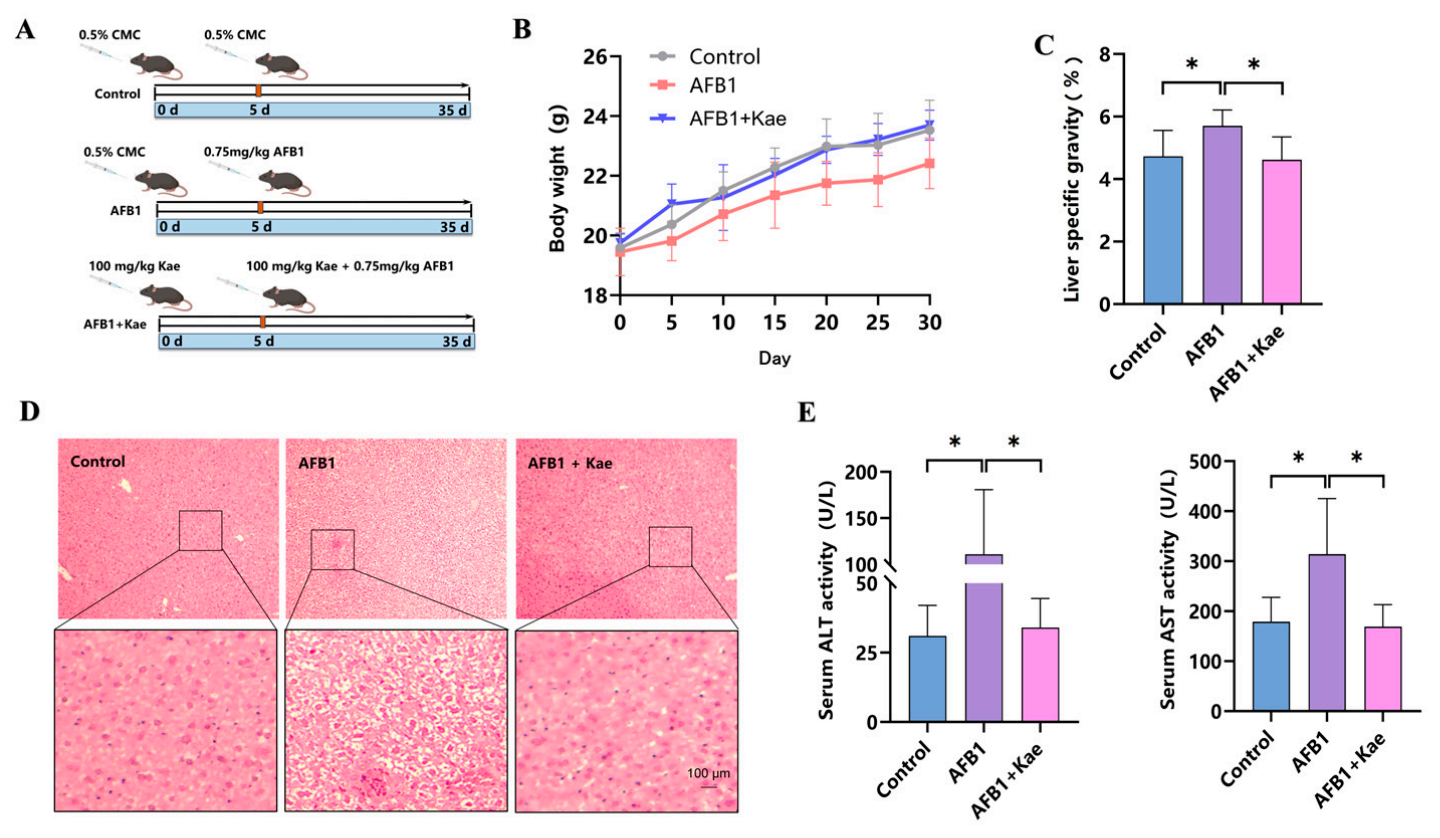
Analysis of body weight and liver injury. (**A**) Schematic diagram of gavage administration. (**B**) Changes in body weight of mice. (**C**) Liver-to-body weight ratio. (**D**) Representative H&E-stained liver sections (scale bar = 100 µm). (**E**) Serum aspartate aminotransferase AST and ALT activity. Significant differences between groups are indicated by asterisks: * *p* < 0.05.

To evaluate the protective effect of Kae against AFB1 hepatotoxicity, a mouse model was established (Figure 1A). Body weight gain was markedly suppressed in AFB1-treated mice compared with controls, whereas Kae co-treatment gradually restored weight gain to near control levels after 20 days (Figure 1B). The liver-to-body weight ratio was significantly higher in the AFB1 group (*p* < 0.05), and Kae reversed this increase (Figure 1C). H&E staining revealed that control mice had clear hepatic lobular architecture, normal hepatocyte morphology, and centrally located nuclei (Figure 1D). In contrast, AFB1-treated livers exhibited severe pathological changes, including hepatocyte necrosis, inflammatory infiltration, and disorganized structure. Kae co-treatment markedly improved these histological alterations. Serum AST and ALT activities were significantly elevated in the AFB1 group (*p* < 0.05), and Kae significantly reduced both enzymes (Figure 1E). Specifically, Kae treatment decreased serum ALT by 69.4% (95% CI: −150.1 to −3.9 U/L, *p* < 0.05) while reducing AST by 46.2% (95% CI: −262.3 to −27.7 U/L, *p* < 0.05) relative to the AFB1 group. These observations lend strong support to the hypothesis that Kae can attenuate AFB1-induced liver injury.

### 3.2. Effect of Kae on AFB1-Induced Hepatic Oxidative Stress

**Figure 2:**
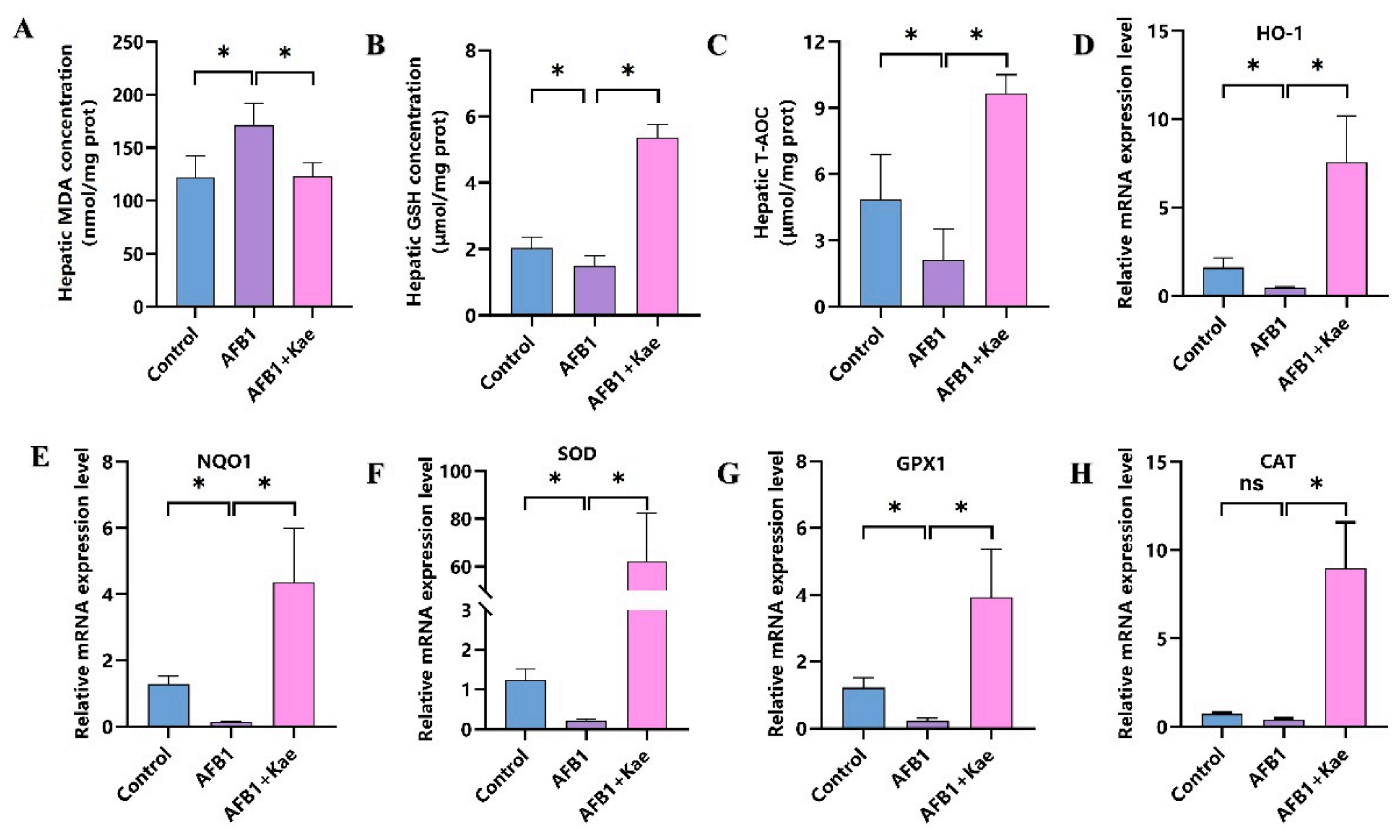
Analysis of hepatic antioxidant capacity. (**A**) MDA content in the liver. (**B**) GSH content in the liver. (**C**) T-AOC content in the liver. (**D**–**H**) Relative mRNA expression levels of *HO-1*, *NQO1*, *SOD*, *GPX1* and *CAT*. Significant differences between groups are indicated by asterisks: * *p* < 0.05. ns indicates no statistically significant difference between groups.

To explore whether Kae could mitigate AFB1-induced hepatic oxidative stress in mice, we assessed relevant oxidative stress markers. The findings revealed that, relative to the control group, MDA content was markedly elevated, whereas GSH level and T-AOC were significantly reduced in the AFB1 group (*p* < 0.05) (Figure 2A–C). RT-qPCR results revealed that Kae treatment significantly upregulated hepatic mRNA levels of HO-1, NQO1, SOD, GPX1, and CAT relative to the AFB1 group (*p* < 0.05) (Figure 2D–H). Collectively, these results demonstrate that Kae strengthens the body’s antioxidant defense by enhancing the transcriptional expression of antioxidant genes, thus alleviating AFB1-elicited oxidative injury.

### 3.3. Network Pharmacology Analysis

**Figure 3:**
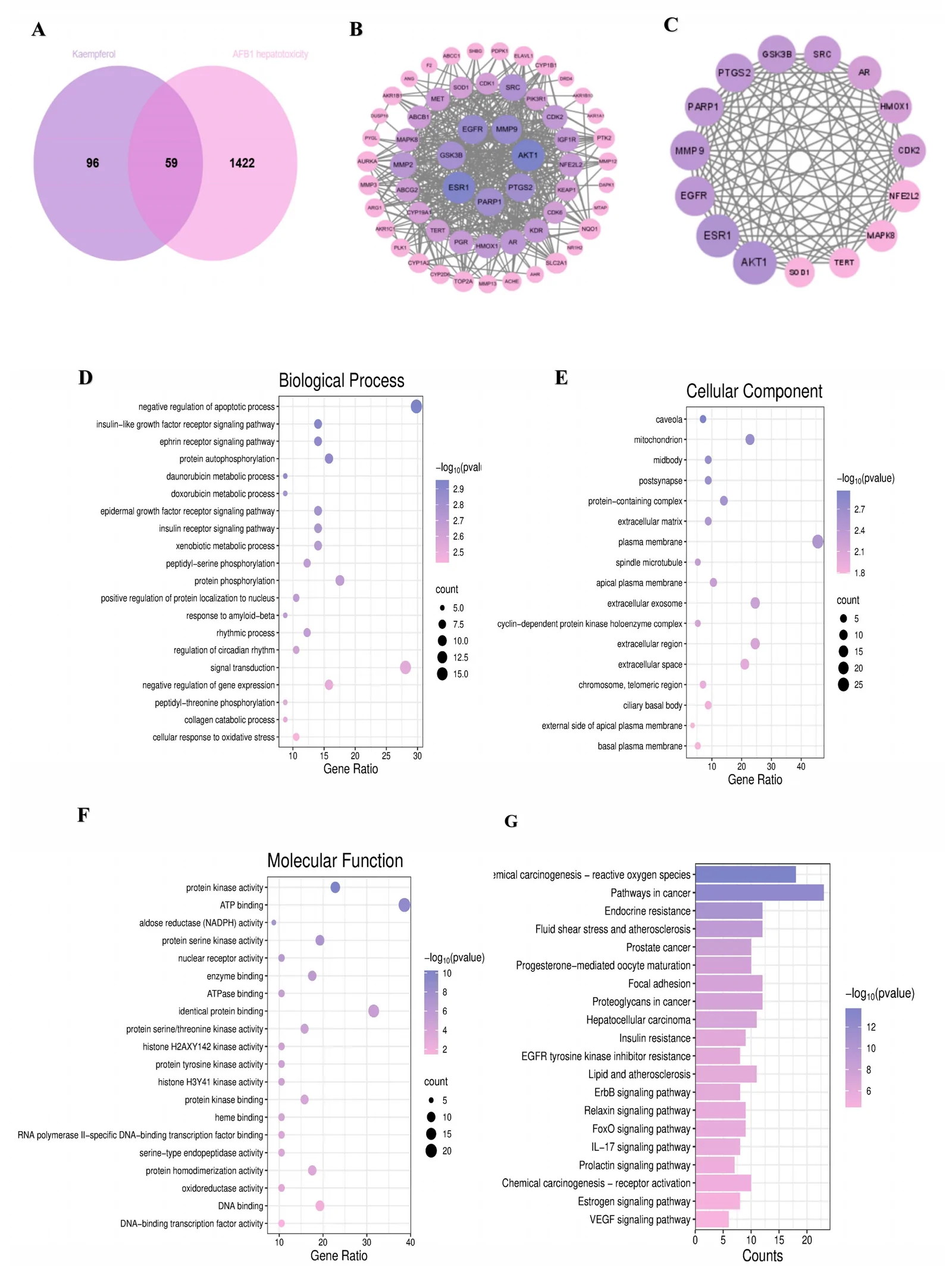
Network pharmacology analysis. (**A**) Venn diagram showing the intersecting targets of kaempferol (Kae) and aflatoxin B1 (AFB1) hepatotoxicity. (**B**) Protein–protein interaction (PPI) network of potential therapeutic targets. (**C**) Core targets. (**D**–**F**) GO enrichment analysis. (**G**) KEGG enrichment analysis.

Given the in vivo evidence that Kae protects against AFB1-induced liver injury in mice, we adopted a network pharmacology strategy to further explore the mechanisms by which Kae alleviates AFB1 hepatotoxicity. A total of 96 targets related to Kae and 1422 targets linked to AFB1 hepatotoxicity were obtained, with 59 overlapping targets (Figure 3A). To systematically clarify how Kae mitigates AFB1-induced liver damage, these 59 shared targets were analyzed using the STRING database, and a protein–protein interaction (PPI) network was constructed after excluding disconnected nodes (Figure 3B). The network was then imported into Cytoscape 3.9.1 for further evaluation. By employing the CentiScaPe 2.2 plugin, degree (DC), betweenness (BC), and closeness (CC) centralities were calculated, leading to the identification of 15 core targets: AKT1, ESR1, EGFR, MMP9, PARP1, PTGS2, GSK3B, SRC, AR, HMOX1, CDK2, NFE2L2, MAPK8, TERT, and SOD1 (Figure 3C).

GO enrichment was performed on the 59 shared targets (Figure 3D–F), covering three functional categories: biological process (BP), cellular component (CC), and molecular function (MF). BP terms were mainly enriched in oxidative stress response, ROS metabolism, apoptosis regulation, and xenobiotic metabolism. CC mapping localized targets to the cytosol, mitochondrion, and protein-containing complexes. MF analysis highlighted ATP binding, nicotinamide adenine dinucleotide phosphate reduced (NADPH) activity, and serine/threonine/tyrosine protein kinase activities (Figure 3D–F). KEGG pathway enrichment analysis (Figure 3G) revealed that the core targets were predominantly enriched in pathways such as chemical carcinogenesis—reactive oxygen species, pathways in cancer, EGFR tyrosine kinase inhibitor resistance, and endocrine resistance.

### 3.4. Effects of Kae on Cell Viability and AFB1-Induced Cellular Injury

**Figure 4:**
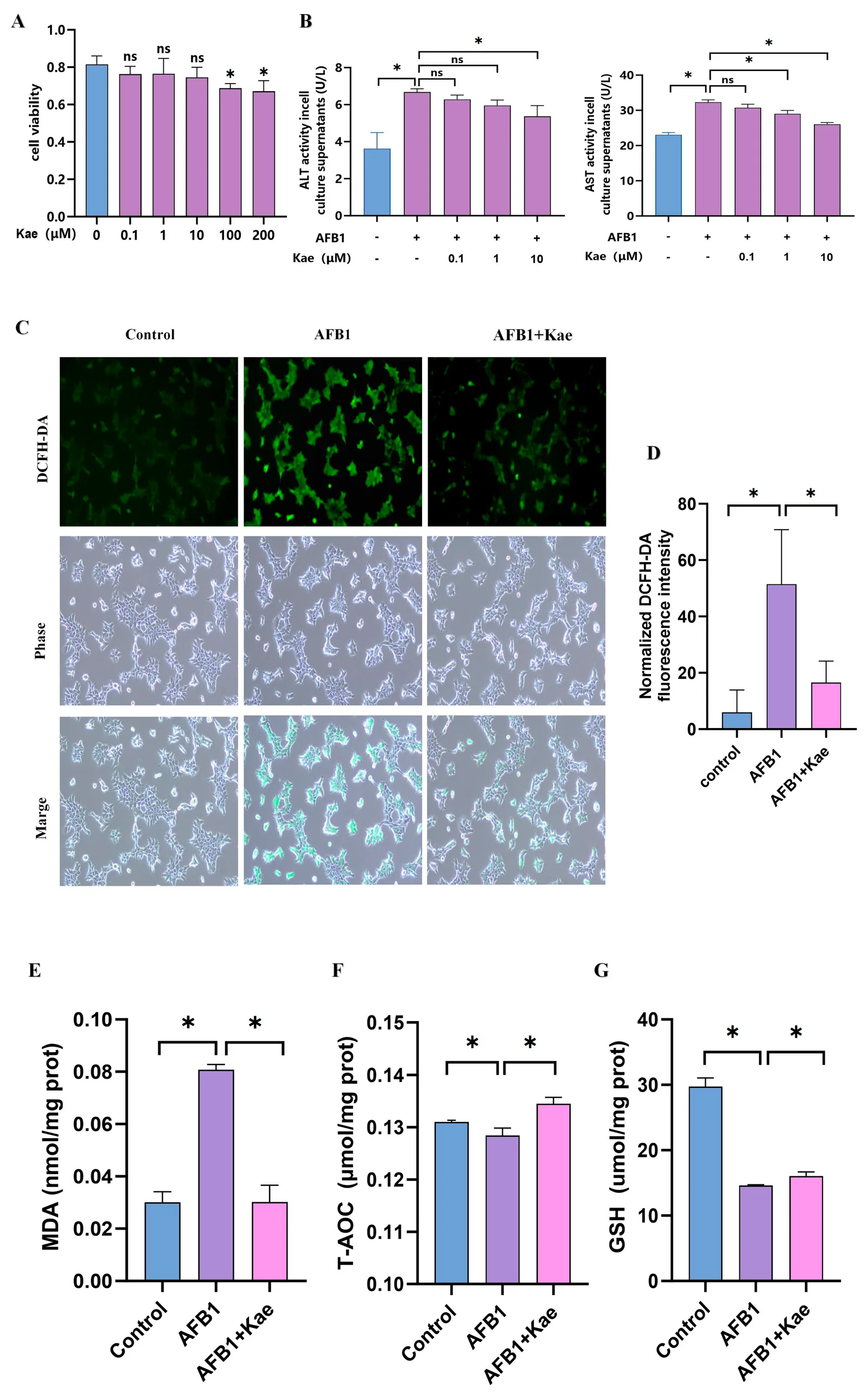
Analysis of cell viability and injury markers. (**A**) Effect of different concentrations of Kae on cell viability. (**B**) Effect of different concentrations of Kae on ALT and AST activity in AFB1-treated AML12 cells. (**C**) Intracellular reactive ROS levels measured by DCFH-DA fluorescent probe method (Control: normal group; AFB1: group treated with Aflatoxin B1; AFB1 + Kae: group co-treated with Aflatoxin B1 and Kae. Green fluorescence indicates the accumulation of reactive oxygen species (ROS)). (**D**) Quantitative graph of intracellular reactive oxygen species levels. (**E**) MDA content. (**F**) T-AOC content. (**G**) GSH content. Significant differences between groups are indicated by asterisks: * *p* < 0.05. ns indicates no statistically significant difference between groups.

To further validate the specific mechanisms at the cellular level, in vitro experiments were subsequently carried out. A CCK-8 assay was used to evaluate the effect of various Kae concentrations (0, 0.1, 1, 10, 100, and 200 μM) on the viability of AML12 cells (Figure 4A). Cell viability was markedly reduced at 100 and 200 μM Kae (*p* < 0.05). We then determined the levels of AST and ALT in AML12 cells. The results showed that pretreatment with 0.1, 1, and 10 μM Kae significantly suppressed the AFB1-induced elevations of AST and ALT in a dose-dependent manner. The most pronounced inhibitory effect was observed in the 10 μM Kae treatment group (*p* < 0.05), with transaminase levels recovering to near those of the normal control group (Figure 4B). Therefore, 10 μM Kae was selected for subsequent experiments.

### 3.5. Kae Attenuates AFB1-Induced Oxidative Stress in Mouse Hepatocytes

To examine whether Kae mitigates oxidative stress in hepatocytes, intracellular ROS production in AML12 cells was detected using the DCFH-DA probe. AFB1-treated cells displayed green fluorescence, reflecting elevated intracellular ROS levels. In contrast, the fluorescence intensity was notably diminished in the AFB1 + Kae group (Figure 4C,D), suggesting that Kae suppresses AFB1-induced ROS generation. We then quantified oxidative stress markers in the cells. In the AFB1 group, MDA content was significantly increased (*p* < 0.05), while GSH and T-AOC levels were significantly decreased (*p* < 0.05). These alterations were reversed by Kae treatment (Figure 4E–G). Taken together, these in vitro findings demonstrate that Kae robustly mitigates AFB1-induced oxidative stress in AML12 hepatocytes.

### 3.6. Kae Alleviates AFB1-Induced Oxidative Stress by Activating the Keap1/Nrf2 Signaling Pathway

**Figure 5:**
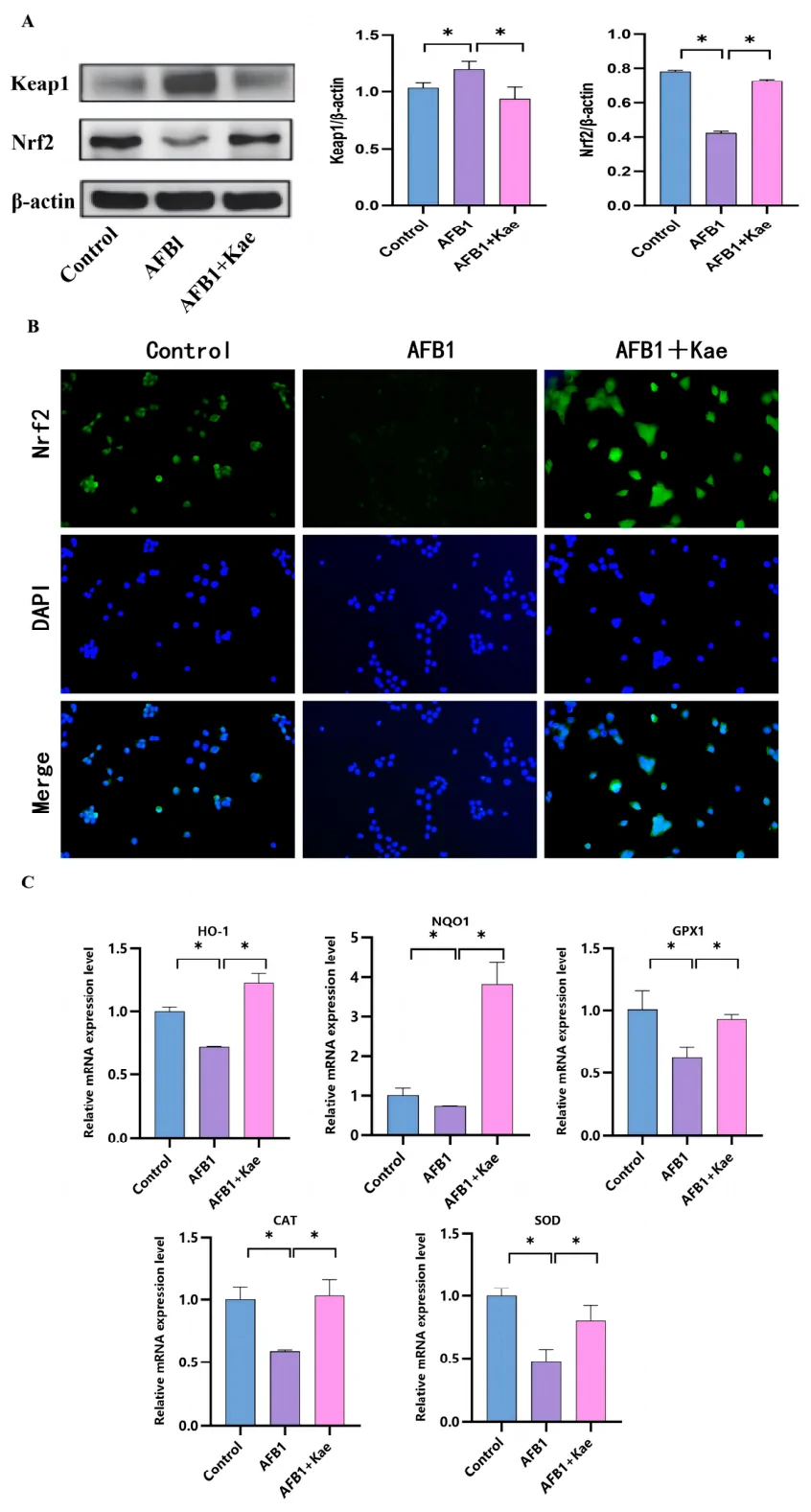
Kae alleviates AFB1-induced oxidative stress by activating the Keap1/Nrf2 signaling pathway. (**A**) Protein expression levels of Keap1 and Nrf2 in AML12 cells. (**B**) Immunofluorescence staining of Nrf2 in AML12 cells, Cells were stained for Nrf2 (green), and nuclei were counterstained with DAPI (blue). Merged images show the colocalization of Nrf2 in the nucleus (cyan). (**C**) Relative mRNA expression levels of *HO-1*, *NQO1*, *GPX1*, *CAT* and *SOD* in AML12 cells. Significant differences between groups are indicated by asterisks: * *p* < 0.05.

To investigate the protective role of Kae against AFB1-induced oxidative injury in AML12 cells, the protein levels of Nrf2 and Keap1 were assessed by Western blotting. Compared with the control group, the AFB1 group exhibited a marked increase in Keap1 protein expression (*p* < 0.05) (Figure 5A) and a significant decrease in Nrf2 protein expression (*p* < 0.05). Notably, Kae treatment reversed these changes. Immunofluorescence staining further confirmed that AFB1 reduced Nrf2 fluorescence intensity and impaired its nuclear translocation, while Kae pretreatment enhanced Nrf2 nuclear accumulation (Figure 5B). Subsequently, we assessed antioxidant gene transcription in AML12 hepatocytes via RT-qPCR. Relative to the control cohort, AFB1 exposure significantly suppressed mRNA levels of *HO-1*, *NQO1*, *GPX1*, CAT, and *SOD* (*p* < 0.05). Strikingly, Kae pretreatment markedly restored their expression (*p* < 0.05) (Figure 5C). These findings indicate that Kae may activate the Nrf2 pathway by interacting with Keap1, thereby promoting Nrf2 protein expression and upregulating the transcription of downstream antioxidant genes, which in turn strengthens cellular antioxidant capacity and ultimately alleviates AFB1-induced oxidative stress.

### 3.7. Molecular Docking and Molecular Dynamics Simulation

**Figure 6:**
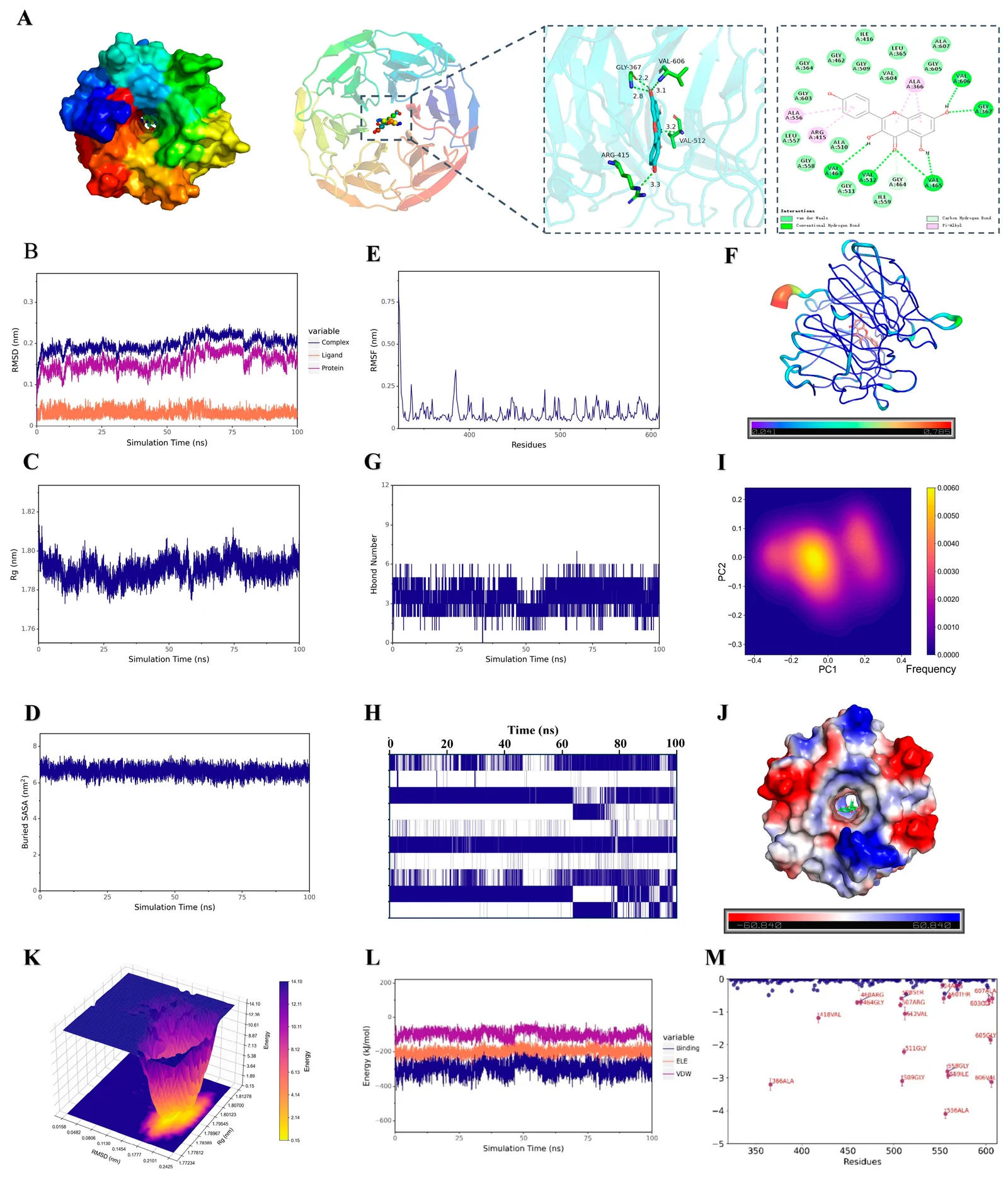
Molecular docking and molecular dynamics simulation of kaempferol (Kae) and Kelch-like ECH-associated protein 1 (Keap1). (**A**) Molecular docking of Kae with Keap1 and visualization. (**B**) RMSD. (**C**) Rg. (**D**) SASA. (**E**) RMSF. (**F**) B-factor. (**G**) Number of hydrogen bonds. (**H**) Frequency of hydrogen bond formation. (**I**) PCA. (**J**) Surface electrostatic potential of the small molecule bound to the protein (unit: kcal/mol). (**K**) FEL. (**L**) VDW and ELE binding energies between the small molecule and the protein. (**M**) Per-residue binding energy contribution.

To verify whether the regulation of the Keap1/Nrf2 pathway by Kae originates from direct molecular interactions, this study employed molecular docking to analyze the binding mode between Kae and the Keap1 protein. The results revealed that Kae stably bound to Keap1 primarily through hydrogen bonds, with key residues including five amino acids such as VAL-606 and GLY-367; additionally, residues such as ILE-559 and GLY-603 further stabilized the complex via van der Waals interactions (Figure 6A). An extensive interaction network was observed overall, indicating the formation of a stable binding conformation between Kae and Keap1. The docking binding energy was −9.6 kcal/mol, suggesting a strong intermolecular affinity. These results provided direct molecular evidence that Kae regulates the Nrf2 antioxidant pathway by targeting Keap1.

To further evaluate the binding stability between Keap1 and Kae, a 100 ns molecular dynamics simulation was performed based on the initial complex conformation obtained from molecular docking. Root-mean-square deviation (RMSD) analysis indicated that the Keap1 protein completed initial conformational adjustments within the first 25 ns, after which the RMSD values stabilized within the range of 0.10–0.17 nm; the ligand RMSD fluctuated between 0.01 and 0.03 nm and gradually approached 0 as the simulation progressed, indicating that the ligand remained stable within the binding pocket and was tightly bound to the protein. The RMSD of the overall complex fluctuated between 0.14 and 0.20 nm without a sustained upward trend, indicating that the complex had reached a stable conformation during the simulation (Figure 6B). Radius of gyration (Rg) analysis showed that the Rg value of the Keap1 protein remained consistently between 1.78 and 1.81 nm, reflecting a highly stable structure (Figure 6C). Buried solvent accessible surface area (SASA) analysis revealed that the binding interface remained stable throughout the simulation, with the buried surface area maintained within the range of 6.6–7.2 nm^2^ (Figure 6D). The stable hydrophobic interaction interface further confirmed the reliability of the complex conformation, collectively supporting the formation of a stable and robust binding mode between Kae and the Keap1 protein, in line with the RMSD and Rg results.

Root-mean-square fluctuation (RMSF) analysis showed that the first five residues exhibited significant fluctuations (up to 0.75 nm), whereas the remaining residues (6–125) were extremely stable (approximately 0.01 nm), indicating high flexibility at the protein termini and strong rigidity of the core structure (Figure 6E). The B-factor plot constructed from the RMSF values of residues surrounding the ligand displayed low flexibility, further confirming the stable binding of the ligand to the protein (Figure 6F). Hydrogen bond analysis revealed that the number of hydrogen bonds between the ligand and the protein was maintained at 2–5 throughout the simulation (Figure 6G). Notably, the hydrogen bond between residue VAL-606 and the ligand exhibited an occupancy frequency of 87.5% (Figure 6H, Table 1), playing a critical role in maintaining binding stability. Principal component analysis (PCA) demonstrated that the conformational distribution of the ligand was relatively concentrated, with two prominent conformational clusters, indicating a substantially stable conformation (Figure 6I). Surface electrostatic potential analysis showed that the ligand bound within a groove on the protein, with the binding site surface predominantly displaying a negative charge (Figure 6J), which favored electrostatic interactions and hydrogen bond formation. Free energy landscape (FEL) analysis indicated the existence of a low-energy state for the complex (Figure 6K), further corroborating the stability of its overall structure.

As the simulation proceeded, both van der Waals (VDW) and electrostatic (ELE) interaction energies gradually stabilized (Figure 6L), suggesting that the ligand–protein binding approached equilibrium. The binding affinity was then quantitatively assessed using the MM-PBSA method. The results revealed that the van der Waals energy (ΔEvdw) was greater than the electrostatic energy (ΔEele), and ΔEele was comparable to the nonpolar solvation energy (ΔEnonpol) (Table 2), indicating that van der Waals forces contributed predominantly to the binding energy, while electrostatic and hydrophobic interactions played supporting roles. Per-residue binding energy decomposition further identified critical amino acid residues, including ALA-556 and ALA-366 (Figure 6M).

In summary, Kae formed a stable and robust binding mode with the Keap1 protein, providing theoretical support for its potential as a Keap1 inhibitor.

## 4. Discussion

This work refines the current understanding of kaempferol’s hepatoprotective mechanisms by linking in silico predictions with experimental validation. While earlier investigations largely emphasized phenotypic endpoints, our results elucidate a mechanistic pathway in which kaempferol directly engages the Keap1/Nrf2 signaling axis, thus supplying a structural basis for its antioxidant activity against AFB1.

AFB1 is a highly toxic mycotoxin that seriously threatens livestock health by causing chronic liver damage [23]. Upon entering hepatocytes, AFB1 undergoes metabolic activation by the cytochrome P450 enzyme system, generating highly reactive toxic intermediates that directly trigger massive intracellular accumulation of ROS and initiate an oxidative stress cascade. Excessive ROS attack biological membranes, DNA, and proteins, causing lipid peroxidation, DNA strand breaks, and oxidative protein modifications, ultimately activating hepatocyte apoptosis and inflammatory response pathways; long-term exposure further promotes malignant transformation of hepatocytes [24]. The liver, being the primary metabolic organ, is also the main target of AFB1 toxicity [25]. ALT and AST are hepatocyte-derived enzymes residing in the cytoplasm and mitochondria. Their serum levels rise markedly following hepatocellular damage, making them widely used biomarkers for liver injury assessment in both clinical practice and experimental animals [26]. In our study, Kae pretreatment significantly suppressed AFB1-induced rises in ALT and AST in mice (*p* < 0.05). Through the reduction in serum ALT/AST levels and oxidative stress, Kae-loaded nanoparticles exhibited hepatoprotective activity in a liver cancer cell model [27].

Oxidative stress is one of the important mechanisms underlying AFB1-induced pathological changes [4]. Lipid peroxidation represents the most characteristic pathological feature during oxidative stress. Excessive ROS attacks polyunsaturated fatty acids in cell membranes, initiating chain reactions that ultimately produce malondialdehyde (MDA). As a result, MDA content is widely used as a quantitative biomarker for assessing the extent of lipid peroxidation and the severity of oxidative damage [28]. GSH is the most abundant non-enzymatic antioxidant in cells; it not only directly scavenges free radicals but also participates in detoxification metabolism and defends against the invasion of exogenous toxins, serving as a key molecule in maintaining cellular homeostasis [29]. T-AOC reflects the sum of cooperative activities from all antioxidants and antioxidant enzymes, making it a valuable marker of overall defense capacity. In essence, higher T-AOC indicates a greater ability to eliminate ROS and protect against oxidative damage [30]. In this study, AFB1 exposure significantly disrupted the redox balance in the mouse liver, as evidenced by decreased hepatic GSH content and T-AOC levels, along with elevated lipid peroxidation product MDA. Meanwhile, the transcript levels of key antioxidant genes such as *HO-1*, *NQO1*, *SOD*, *GPX1*, and *CAT* were significantly suppressed. Following Kae intervention, all these indicators were markedly improved, and the hepatic redox imbalance was effectively reversed. In summary, Kae can regulate the body’s antioxidant defense system through multiple targets and attenuate AFB1-induced oxidative stress injury, which constitutes one of the important molecular mechanisms underlying its hepatoprotective effect.

Network pharmacology, a key approach in systems biology, is commonly used to predict the multi-target and multi-pathway actions of natural products [31]. In this study, through multi-database integration and screening, 15 core targets of Kae against AFB1 hepatotoxicity were identified. These targets are extensively involved in signaling networks related to oxidative stress defense, apoptosis and survival, inflammation, DNA damage repair, and cell proliferation. Among the identified core targets are COX2, MMP9, ESR1, and PARP1. Studies have consistently linked the COX2-adrenergic signaling axis to liver fibrosis, hepatocellular carcinoma, and cirrhosis. This pathway exerts its anti-fibrotic actions by inhibiting the ability of hepatic stellate cells to contract, migrate, and proliferate [32]. As a gelatinase of the MMP family, MMP9 degrades type IV collagen, fibronectin, and elastin, thereby promoting liver fibrosis regression and scar remodeling, and thus plays an important role in liver injury repair [33,34]. ESR1 mainly mediates estrogen signaling and influences hepatic metabolism and inflammatory responses [35]. PARP1 is a DNA repair enzyme and a substrate of caspases; caspase-3 and caspase-7 can cleave and inactivate PARP1, thereby triggering apoptosis [36,37]. GO functional enrichment analysis indicated that these targets are primarily enriched in biological processes including signal transduction, apoptosis regulation, and redox reactions. Their molecular functions are mainly associated with protein kinase activity, oxidoreductase activity, and ATP binding. These findings suggest that Kae may counteract AFB1-induced liver injury by modulating multiple cellular physiological processes. KEGG pathway enrichment further showed that the core targets of Kae are mainly enriched in chemical carcinogenesis-ROS, cancer-related pathways, and EGFR tyrosine kinase inhibitor resistance. These results suggest that kaempferol may contribute to the intervention of chemical carcinogenesis by coordinately influencing oxidative stress, xenobiotic metabolism, and apoptotic processes. In vitro experiments further confirmed that in the AML12 hepatocyte model, AFB1-induced oxidative stress injury was successfully replicated. Meanwhile, Kae significantly suppressed the AFB1-triggered ROS burst, reduced ALT and AST release caused by hepatocyte damage, effectively elevated intracellular GSH content and total antioxidant capacity (T-AOC), and decreased the level of the lipid peroxidation end-product MDA. Although the representative ROS fluorescence images provided a visual indication of oxidative status, the quantitative biochemical and molecular analyses presented herein offer a more rigorous evaluation of Kae’s antioxidant efficacy. This protective effect of Kae is consistent with the finding of Dai et al. [38] that curcumin protects against AFB1-induced liver injury, indicating that natural bioactive components alleviate AFB1-induced liver injury by ameliorating oxidative stress.

Nrf2 (nuclear factor erythroid 2-related factor 2) is a key regulator encoded by the NFE2L2 gene. It plays a central role in protecting the body against external toxins and in preserving redox balance [39]. Under basal homeostatic conditions, the transcription factor nuclear factor erythroid 2-related factor 2 (Nrf2) is sequestered in the cytosolic compartment through its physical association with Kelch-like ECH-associated protein 1 (Keap1). This protein–protein interaction licenses Keap1-mediated conjugation of ubiquitin moieties to Nrf2, targeting the transcription factor for continuous proteasomal turnover. Upon exposure to reactive oxygen species or electrophilic xenobiotics, evolutionarily conserved reactive cysteine residues within the Keap1 sensor domain undergo covalent modification, triggering a large-scale conformational rearrangement of the repressor complex. This structural perturbation dissociates Nrf2 from its cytoplasmic repressor, permitting nuclear translocation of the liberated transcription factor, where it binds to antioxidant response element (ARE) in promoter regions of target genes to drive transcription of cytoprotective enzymes and restore redox homeostasis [40,41,42]. In a cellular model of rotenone-induced Parkinsonian-like damage, resveratrol-loaded polymeric nanoparticles exert protective effects by decreasing ROS, sustaining mitochondrial function, and limiting apoptosis, all mediated via the Nrf2/HMOX-1 pathway [43]. Daily consumption of cashew nuts can combat oxidative stress by regulating the Nrf2 antioxidant pathway, which in turn lowers lipid peroxidation and increases antioxidant enzyme activity [44]. Cao et al. [45] found that arginine-derived carbon dots upregulated the transcriptional activities of Nrf2 and its downstream antioxidant effectors and phase II detoxifying enzymes, thereby alleviating AFB1-induced liver injury in mice. Based on the network pharmacology prediction that *NFE2L2* is one of the core targets of Kae against AFB1 hepatotoxicity, we hypothesized that the specific mechanism by which Kae alleviates AFB1-induced liver injury may be mediated through the Keap1/Nrf2 pathway. We therefore validated this hypothesis through in vitro experiments. Western blot analysis revealed that Kae treatment significantly reversed the AFB1-induced upregulation of Keap1 protein expression and downregulation of Nrf2 protein expression. Concurrently, RT-qPCR results showed that Kae upregulated the mRNA levels of downstream target genes such as *HO-1* and *NQO1*. These findings suggest that activation of the Nrf2 antioxidant pathway may be the molecular mechanism by which Kae counteracts AFB1-induced oxidative damage in hepatocytes.

Although network pharmacology predicted Nrf2 as a core target, the regulation of this target depends on the upstream protein Keap1. Under normal physiological conditions, Nrf2 is bound by Keap1 and anchored in the cytoplasm, where it is degraded via the ubiquitination pathway to maintain low levels. The regulation of Nrf2 activity is primarily achieved by relieving Keap1-mediated ubiquitination and degradation [46]. Under physiological and pharmacological conditions, most natural products do not directly bind to the Nrf2 protein and induce its conformational change; instead, they “release” Nrf2 by competing for the Kelch domain of Keap1 to disrupt the Keap1-Nrf2 interaction [47]. In vitro experiments have demonstrated that Kae promotes Nrf2 expression; therefore, we hypothesized that Kae may bind to the Kelch domain of Keap1 to block the Keap1-Nrf2 interaction, thereby activating Nrf2. Accordingly, we performed molecular docking between Kae and Keap1. The molecular docking experiment verified the interaction between Kae and the Keap1 protein, with a docking score of −9.6 kcal/mol. A lower binding score indicates greater binding stability between the small molecule and the target protein, suggesting a strong intermolecular interaction and favorable binding specificity between Kae and Keap1. The molecular docking results preliminarily revealed the binding mode between Kae and Keap1; however, static docking cannot fully reflect the dynamic binding characteristics of the complex under physiological conditions. It is important to note that molecular docking and dynamics simulations are computational approaches that predict binding affinity and stability; while they provide robust theoretical support for the interaction between Kae and Keap1, these findings should be validated by direct biophysical experiments (e.g., Surface Plasmon Resonance or Isothermal Titration Calorimetry) in future studies. Therefore, this study further conducted a 100 ns molecular dynamics simulation to evaluate the dynamic stability of the Kae–Keap1 complex. The results demonstrated that Kae formed a highly stable complex with the Keap1 protein, driven primarily by van der Waals forces and the key hydrogen bond with VAL-606, with a compact binding interface and low flexibility. Binding energy decomposition further identified key residues such as ALA-556 and ALA-366, providing a theoretical basis for understanding the molecular mechanism of Kae as a potential Keap1 inhibitor. Previous studies have similarly found that Asp-Trp-Ser maintains conformational stability by forming hydrogen bonds with VAL-606 of Keap1 [48]. The dominance of van der Waals forces in the binding energy observed in this study is consistent with the results reported by Guo et al. [49] for the Keap1–Seq10 complex. Furthermore, Chen et al. [50] screened multiple natural products from *Achyranthis bidentatae* Radix that directly inhibited Keap1-Nrf2 protein–protein interactions through hydrophobic interactions, hydrogen bonds, and salt bridges, which aligns with the binding mode of Kae observed in this study. The above results corroborate the molecular docking findings, collectively supporting the formation of a stable and robust binding mode between Kae and Keap1.

While this study clarifies kaempferol’s protective mechanism against AFB1 hepatotoxicity, key limitations warrant note. Firstly, the small sample size (*n* = 6/group) and single male mouse model limit generalizability; we also lacked Nrf2 inhibition/knockdown validation, and treated molecular docking results as predictive rather than definitive. Secondly, unaddressed phase II metabolism and the low (2–5%) oral bioavailability of parent kaempferol constrain direct clinical translation, underscoring the need for metabolite-focused studies or nano-formulation development.

## 5. Conclusions

In conclusion, this study demonstrated through multi-level experiments that Kae can effectively alleviate AFB1-induced oxidative stress-mediated liver injury by scavenging free radicals and activating the Keap1/Nrf2 signaling pathway, upregulating the expression of downstream antioxidant genes, and enhancing the body’s antioxidant defense capacity. This finding not only elucidates the molecular mechanism by which Kae mitigates AFB1 hepatotoxicity but also provides novel insights for developing AFB1 detoxification agents based on natural flavonoids, offering significant theoretical value and application prospects.

## Figures and Tables

**Table 1:** Hydrogen bond pairs between small molecules and proteins.

Serial Number	Receptor	Donor
1	367GIY(chain_A#N)	Ligand(4360#O)
2	415ARG(chain_A#NE)	Ligand(4359#O)
3	557LEU(chain_A#O)	Ligand(4358#O)
4	559ILE(chain_A#N)	Ligand(4358#O)
5	559ILE(chain_A#N)	Ligand(4361#O)
6	606VAL(chain_A#N)	Ligand(4360#O)
7	Ligand(4360#O)	367GLY(chain_A#O)
8	Ligand(4360#O)	606VAL(chain_A#O)
9	Ligand(4362#O)	510ALA(chain_A#O)
10	Ligand(4362#O)	557LEU(chain_A#O)

**Table 2:** Binding energy and its composition under stable state (unit: kJ/mol).

Complex	Protein-Ligand
ΔEvdw	−147.962 ± 2.584
ΔEele	−20.638 ± 2.201
ΔEpol	134.259 ± 3.165
ΔEnonpol	−17.699 ± 0.022
ΔEMMPBSA	−52.04 ± 2.066
−TΔS	5.772 ± 0.637
ΔGbind *	−46.268 ± 2.569
ΔEvdw	−147.962 ± 2.584
ΔEele	−20.638 ± 2.201

Note: * ΔGbind = ΔEvdw + ΔEele + ΔEpol + ΔEnonpol − TΔS.

## Data Availability

All raw data generated during this study are available from the corresponding author upon reasonable request. No publicly archived datasets were used in this work.

## Supplementary Materials

The following supporting information can be downloaded at: https://www.mdpi.com/article/10.3390/nu18162633/s1, Table S1: Primer Information.

## Abbreviations

AFB1	Aflatoxin B1
AFBO	Aflatoxin B1-8,9-epoxide
ALT	Alanine aminotransferase
ARE	Antioxidant response element
AST	Aspartate aminotransferase
BC	Betweenness centrality
BP	Biological process
CAT	Catalase
CCK-8	Cell Counting Kit-8
CMC	Carboxymethylcellulose
COX2	Cyclooxygenase-2
CC	Cellular component/Closeness centrality
CYP	Cytochrome P450
DC	Degree centrality
Rg	Radius of gyration
DCFH-DA	2′,7′-Dichlorodihydrofluorescein diacetate
DAPI	4′,6-Diamidino-2-phenylindole
ECL	Enhanced chemiluminescence
EGFR	Epidermal growth factor receptor
RMSF	Root-mean-square fluctuation
ELE	Electrostatic energy
FBS	Fetal bovine serum
FEL	Free energy landscape
GO	Gene Ontology
GPX1	Glutathione peroxidase 1
GSH	Glutathione (reduced)
H&E	Hematoxylin and eosin
HO-1	Heme oxygenase-1
Keap1	Kelch-like ECH-associated protein 1
Kae	Kaempferol
KEGG	Kyoto Encyclopedia of Genes and Genomes
MDA	Malondialdehyde
MF	Molecular function
MMP9	Matrix metalloproteinase 9
MOLE	Moringa oleifera leaf extract
Nrf2	Nuclear factor erythroid 2-related factor 2
NQO1	NAD(P)H quinone oxidoreductase 1
NVT/NPT	Constant volume/temperature and constant pressure/temperature ensembles
NFE2L2	Nuclear factor erythroid 2-related factor 2 (gene name for Nrf2)
PCA	Principal component analysis
PARP1	Poly(ADP-ribose) polymerase 1
PDB	Protein Data Bank
PPI	Protein–protein interaction
PBS	Phosphate-buffered saline
PME	Particle mesh Ewald
RMSD	Root-mean-square deviation
ROS	Reactive oxygen species
RT-qPCR	Reverse transcription quantitative polymerase chain reaction
SDS-PAGE	Sodium dodecyl sulfate-polyacrylamide gel electrophoresis
SOD	Superoxide dismutase
SASA	Solvent accessible surface area
T-AOC	Total antioxidant capacity
VDW	Van der Waals (energy/forces)
