# Supplementary material for: Kaempferol Alleviates Aflatoxin B1-Induced Liver Injury by Mitigating Oxidative Stress
Source: Nutrients. 2026 Aug 12;18(16):2633. doi: 10.3390/nu18162633 (PMC13516749; doi:10.3390/nu18162633)
Supplement: Supplementary file 1 [file nutrients-18-02633-s001.zip › nutrients-4448290-supplementary.pdf]

Table S1. Primer Information

Gene name	Sequence（5′→3′）	Length (bp)
HO-1	F: CACTCTGGAGATGACACCTGAG	22
	R: GTGTTCCTCTGTCAGCATCACC	22
NQO1	F: GCCGAACACAAGAAGCTGGAAG	22
	R: GGCAAATCCTGCTACGAGCACT	22
SOD	F: CCAGTGCAGGACCTCATTTT	20
	R: AATCCCAATCACTCCACAGG	20
GPX1	F: TGCGGAATGCCTTGCCAACAC	21
	R:AGCCAGTAATCACCAAGCCAATGC	24
CAT	F: CACTGACGAGATGGCACACT	20
	R: TGTGGAGAATCGAACGGCAA	20
β-actin	F:TCTGGCACCACACCTTCTA	21
	R:AGGCATACAGGGACAGCAA	19
